# The Bidirectional Transfer and Fetal Vascular Pressure Changes Due to the Presence of ${}^{125}$I-Labeled Inhibin A in the *ex-vivo* Human Placental Model

**DOI:** 10.1080/10647440300025505

**Published:** 2003

**Authors:** Roger E. Bawdon, Victor Ghetie

**Affiliations:** 1 Department of Obstetrics and Gynecology University of Texas Southwestern Medical Center 5323 Harry Hines Blvd Dallas TX 75390-9032 USA; 2 Cancer Immunobiology Center University of Texas Southwestern Medical Center Dallas TX USA

## Abstract

*Objective:*  The purpose of this study was to investigate the transport of inhibin A and to determine its effects on fetal vascular pressure at elevated levels in the human placenta using ^125^I -labeled synthetic glycoprotein.

Methods: Synthetic inhibinAwas prepared and was shown to be consistent with the natural form by high-pressure liquid chromatography (HPLC) and molecular weight determination by gas-chromatography mass spectrometry. The standardized Na^125^I process yielded ^125^I -labeled inhibin A with a radioactivity of 10^6^ cpm/μg. This compound was placed in the human placenta in maternal–fetal and fetal–maternal studies using antipyrine and ^14^C -labeled inulin as controls to determine the bidirectional transfer of the compound.

Results: Maternal–fetal and fetal–maternal clearance indices were 0.045± 0.003 and 0, respectively. In eight placentas there was no evidence of vascular pressure changes due to the presence of up to 5000 pg of inhibin A.

Conclusions: There is minimal maternal–fetal transfer and no detectable fetal–maternal transfer in normotensive and pregnancy-induced hypertensive placentas. In addition, there are no pressure changes in the fetal vascular system due to the clinically significant levels of inhibin A.


*Infect Dis Obstet Gynecol 2003;11:101–104*

### The bidirectional transfer and fetal vascular pressure changes due to the presence of $^{125}$I-labeled inhibin A in the *ex-vivo* human placental model

**Roger E. Bawdon[1] and Victor Ghetie[2]**

[1]*Department of Obstetrics and Gynecology and*
[2]*Cancer Immunobiology Center, University of Texas Southwestern Medical Center, Dallas, TX*

***Objective:*** The purpose of this study was to investigate the transport of inhibin A and to determine its effects on fetal vascular pressure at elevated levels in the human placenta using $^{125}$I-labeled synthetic glycoprotein.
***Methods:*** Synthetic inhibin A was prepared and was shown to be consistent with the natural form by high-pressure liquid chromatography (HPLC) and molecular weight determination by gas-chromatography mass spectrometry. The standardized Na$^{125}$I process yielded $^{125}$I-labeled inhibin A with a radioactivity of $10^6$ cpm/μg. This compound was placed in the human placenta in maternal–fetal and fetal–maternal studies using antipyrine and $^{14}$C-labeled inulin as controls to determine the bidirectional transfer of the compound.
***Results:*** Maternal–fetal and fetal–maternal clearance indices were $0.045 \pm 0.003$ and 0, respectively. In eight placentas there was no evidence of vascular pressure changes due to the presence of up to 5000 pg of inhibin A.
***Conclusions:*** There is minimal maternal–fetal transfer and no detectable fetal–maternal transfer in normotensive and pregnancy-induced hypertensive placentas. In addition, there are no pressure changes in the fetal vascular system due to the clinically significant levels of inhibin A.

Key words: RADIOLABELED INHIBIN A; MATERNAL–FETAL TRANSFER; FETAL–MATERNAL TRANSFER; FETAL PRESSURE

---

Inhibin is a dimeric, disulfide-linked glycoprotein that is produced in the ovaries and the placenta, and it is probably involved in the stimulation and inhibition of cellular differentiation, proliferation and morphogenesis. During gestation the placenta is the main source of inhibin, the level of which generally rises through gestation and peaks at term. Inhibin consists of two subunits, namely the A fragment and the B fragment. The A fragment contains 33 amino acids with a molecular weight of 3796 daltons, and the inhibin B fragment has 27 amino acids with a molecular weight of 3300 daltons. Further studies indicate that there are at least two forms of inhibin A, namely the biological form and the immunological form, the latter form being reactive in enzyme-linked immunosorbent assays (ELISA) and the former only being responsible for selectively inhibiting the secretion of follicle-stimulating hormone (FSH)[1–4]. The level of inhibin A (probably the immunological form) has been shown to be elevated in patients diagnosed with pre-eclampsia. Previous studies have shown that inhibin A levels become elevated as early as 25 weeks' gestation in pre-eclampsia and gestational hypertension. These levels may exceed a median level of 1833 pg/ml in severe cases[5]. The

Correspondence to: Roger E. Bawdon, PhD, Department of Obstetrics and Gynecology, University of Texas Southwestern Medical Center, 5323 Harry Hines Blvd, Dallas, TX 75390-9032, USA. E-mail: Roger.Bawdon@utsouthwestern.edu

© 2003 The Parthenon Publishing Group 

present study investigated the bidirectional transfer of inhibin A in normotensive and pre-eclamptic placentas, and determined its effect on fetal vessel pressure in the presence of elevated levels of the glycoprotein.

### MATERIALS AND METHODS

The *ex-vivo* human placental model was used in this study, with modification according to Challier[6], Schneider *et al.*[7] and Brandes *et al.*[8]. This model has been used extensively to determine the transfer and pressure effects of numerous drugs, including some antiviral and antimicrobial compounds[9,10]. These studies were approved by the Institutional Review Board of our institution. Eight placentas from Cesarean sections were transported to the laboratory within 10 minutes of delivery. The fetal artery and vein of a suitable cotyledon were cannulated with 3° and 5° French catheters, respectively, to establish a 'fetal circulation'. The maternal circulation was established by insertion of two 18-guage blunt needles about 1 cm into the blanched intervillous space of the selected cotyledon. Both the maternal and fetal circulations were perfused with heparinized drug-free Eagles minimum essential medium containing 3% bovine serum albumin. After approximately 30 minutes of perfusion, and if no fetal–maternal leaks were detected, the flow rates were set at 5.0 and 17.0 ml/minute, respectively. The perfusates were maintained at 37°C with the system in a closed transparent container. The pH was 7.35, and 95% $O_2$ and 5% $CO_2$ were bubbled into the perfusates to maintain physiological pH and $O_2$ concentration. Inhibin A was synthesized by a Ranin Symphony Automated Peptide Synthesizer and was provided by the Protein Technology Center at the Howard Hughes Medical Institute, University of Texas Southwestern Medical Center. Molecular weight determinations were done by gas-chromatography (GC) mass spectrometry, and purity was confirmed by high-pressure liquid chromatography (HPLC). This was in order to confirm that inhibin A was produced by this methodology.

### Iodination of inhibin A

Inhibin (100 μg) dissolved in 100 μl of 0.1 mol/l carbonate–bicarbonate buffer, pH 9.0, was radiolabeled with 1 mCi of $Na^{125}I$ using the Iodogen reagent (Amersham, Arlington Heights, IL)[11,12]. The free iodine was removed by chromatography on a microcolumn of Amberlite IRA-400 (Fluka, Switzerland) equilibrated with the above-mentioned buffer. The specific radioactivity of the $^{125}I$-labeled inhibin A was around $10^6$ cpm/μg.

Samples from the maternal and fetal circulations were collected every 10 minutes over a period of several hours. One ml of sample was placed in a 12 mm × 75 mm test tube, and 1.0 ml of 10% trichloroacetic acid (TCA) was then added and the sample was vortexed. The samples were centrifuged at 2000 rpm for 10 minutes to pellet the precipitated radiolabeled inhibin and other protein; this allows the removal of free iodine. The pellets were washed twice with 10% TCA and counted on a gamma counter for 1 minute (Packard United Technologies Minaxi Auto-Gama 5000 Series, Dowers Grove, IL).

### Antipyrine

Approximately 100 μg/ml of antipyrine was added to the maternal circulation of each placenta. Antipyrine concentrations were determined using HPLC. In total, 0.5 ml of maternal or fetal samples was extracted with 10% TCA for protein and blood precipitation. The samples were centrifuged for 10 minutes at 2000 × g, and 20 μg of aqueous layer were injected into the HPLC system. The HPLC conditions included a 486 detector, a 712 Waters Intelligent Sample Processor, a $C_{18}$ u-bond-a-pak column and a 10 mV recorder (Waters Instruments, Milford, MA). The detector sensitivity was 0.5 absorbance units at a wavelength of 254 μm. The flow rate of the 25% acetonitrile in 0.01 mol/l $KPO_4$ buffer, pH 7.1, was 2.0 ml/minute.

### Determination of $^{14}C$-labeled inulin

Inulin was added to the fetal circulation at a concentration of 60 mg/l. $^{14}C$-labeled inulin was added to the fetal media with a specific activity of 15.5 μCi/μg. $^{14}C$-labeled inulin was determined by the addition of 0.10 ml of maternal or fetal sample to a scintillation vial, then adding 5.0 ml of counting cocktail and counting on a liquid scintillation counter (Pharmacia Wallac Liquid Scintillation Counter, Perkin Elmer Life Science Corp., Boston, MA).

### RESULTS

At 5000 pg/ml, the maternal–fetal clearance index (CI) of $^{125}I$-labeled inhibin A was $0.045 \pm 0.003$, indicating little or no transfer of the labeled compound. The fetal–maternal CI of $^{125}I$-labeled inhibin A at concentrations of up to 5000 pg/ml was undetectable. Furthermore, the maternal– fetal CI of the compound was 0.09 in the two pregnancy-induced hypertensive placentas, which was in the range of that for the four normal placentas. There appears to have been no change due to pregnancy-induced hypertension. In addition, with these high levels of inhibin A there was no change in the fetal vascular pressure such as has been observed in previous placental perfusion studies with antihypertensive drugs[13].

### DISCUSSION

In an effort to determine the movement of inhibin A in the maternal–fetal compartment, $^{125}I$-labeled inhibin A was used at elevated concentrations not only to investigate this transfer but also to determine the effects on the vascular pressure in the fetal vessels. $^{125}I$-labeled inhibin A was used not only to distinguish the compound from the existence of endogenous inhibin A, but also to allow an extremely sensitive radiolabeling methodology to be used. It must be noted that the synthetic peptide produced by Sigma (St Louis, MO) and the peptide that was synthesized in our laboratory did not react with the ELISA amplification kit. Although the Sigma peptide did inhibit the secretion of FSH in luteal cells, our peptide was not tested. This finding may be due to the addition of tyrosine, which is essential for binding the peptide to $^{125}I$, thus perhaps changing the receptor site used in the ELISA amplification kit. However, it may also be a biological rather than an immunological form of the peptide that reacts in the ELISA method[11,12,14].

Furthermore, with regard to the nonreactivity with the ELISA amplification kit of our peptide and that prepared by Sigma, both compounds were identical as assessed by GC mass spectrometry, and the results of molecular weight determinations and HPLC analyses were identical. There may be an apparent difference between the biological and immunological forms of inhibin A. In addition, there are possible artifacts of the radiolabeled inhibin that may interfere with the ELISA-A assay[2,3]. Further studies indicate that cord serum interferes with the measurement of human inhibin A, due to the presence of inhibin-binding proteins or proteolytic enzymes[3,4].

As has been described in earlier publications, inhibin A is not present as such in the fetal circulation, and as our study demonstrates, it is not transferred to the maternal circulation. In addition, maternal–fetal transfer is minimal in the *ex-vivo* system, and is not related to an increase in vascular pressure.

### COMMENTS

The potential flaw in this study is the lack of reactivity with the inhibin in the ELISA-A assay kit. The nonreactivity of the ELISA kit may be due to the addition of the amino acid tyrosine for the radioactive iodination.

### REFERENCES

1. Kettel LM, Roseff SJ, Bangah ML, *et al*. Circulating levels of inhibin in pregnant women at term: simultaneous disappearance with oestradiol and progesterone after delivery. *Clin Endocrinol* 1991; 34:19–23
2. Billiar RB, Smith P, Falcone T. Identification of immunoreactive inhibin in human and baboon fetal serum at term as free alpha-subunits. *J Clin Endocrinol Metab* 1995;80:3173–9
3. Bramley TA, Menzies GS, Baxter G, *et al*. Apparent alpha-inhibin subunit immunoactivity in porcine and ovine luteal extracts is due to interference by cytosolic proteases in the assay. *J Endocrinol* 1992;134:341–52
4. Krummen LA, Woodruff TK, DeGuzman G, *et al*. Identification and characterization of binding proteins for inhibin and activin in human serum and follicular fluids. *Endocrinology* 1993;132:431–3
5. Silver HM, Lambert-Messerlian GM, Star JA, *et al*. Comparison of maternal serum total activin A and inhibin A in normal, pre-eclamptic and nonproteinuric gestationally hypertensive pregnancies. *Am J Obstet Gynecol* 1999;180:1131–7
6. Challier JC. Criteria for evaluating perfusion experiments and presentation of results. *Contrib Gynecol Obstet* 1985;13:32–9
7. Schneider H, Panigel M, Dancis J. Transfer across the perfused human placenta of antipyrine, sodium and leucine. *Am J Obstet Gynecol* 1972;114:822–8
8. Brandes JM, Tavoloni N, Potter BJ, *et al*. A new recycling technique for human placental cotyledon perfusion: applications to studies of the feto-maternal transfer of glucose, inulin and antipyrine. *Am J Obstet Gynecol* 1983;146:800–6
9. Bawdon RE. The *ex-vivo* human placental transfer of the anti-HIV nucleoside inhibitor abacavir and the protease inhibitor amprenavir. *Infect Dis Obstet Gynecol* 1998;6:244–6
10. Casey B, Bawdon RE. *Ex-vivo* human placental transfer of trovafloxacin. *Infect Dis Obstet Gynecol* 2000;8:228–9
11. Yamashiro D, Li CH, Ramasharma K, *et al*. Synthesis and biological activity of human inhibin-like peptide (1-31). *Proc Natl Acad Sci USA* 1984;81:5399–402
12. Kim JK, Tsen MF, Ghetie V, *et al*. Identifying amino acid residues that influence plasma clearance of murine IgG1 fragments by site-directed mutagenesis. *Eur J Immunol* 1994;24:542–8
13. Magee KP, Bawdon RE. *Ex-vivo* human placental transfer and vasoactive properties of hydralazine. *Am J Obstet Gynecol* 2000;182:167–9
14. Sairam MR, Ramasharma K, Li CH. Synthetic peptide with inhibin-like activity preferentially inhibits follitropin secretion in comparison with lutropin-releasing hormone antagonists. *Proc Natl Acad Sci USA* 1987;84:2043–6

RECEIVED 08/30/02; ACCEPTED 09/25/02
